# The impact of mental health literacy on depression, anxiety and well-being among vocational nursing students: mediating roles of resilience

**DOI:** 10.3389/fpsyt.2025.1585642

**Published:** 2025-06-30

**Authors:** Yanying Yang, Lihong Miao, Xuejuan Liu, Weicai Su, Sujiao Liu

**Affiliations:** ^1^ Department of Nursing, Henan Vocational College of Nursing, Anyang, Henan, China; ^2^ Medical Care School, Chengdu Polytechnic, Chengdu, Sichuan, China; ^3^ School of Nursing, Changji Vocational and Technical College, Changji, Xinjiang, China; ^4^ Department of Nursing, Beijing Health Vocational College, Beijing, China

**Keywords:** mental health literacy, resilience, well-being, anxiety/depression, nursing students, cross-sectional

## Abstract

**Background:**

Anxiety and depression are increasingly prevalent among nursing students. Mental Health Literacy (MHL) has been regarded as a potential protective factor for mental health. However, the relationship between MHL, resilience, anxiety/depression and well-being among nursing students is still understudied.

**Objective:**

This study aimed to explore the relationship between MHL and anxiety, depression and well-being, as well as the mediating role of resilience among nursing students.

**Methods:**

We adopted a cross-sectional online questionnaire approach using the “Questionnaire Star” platform. The Mental Health Literacy Questionnaire-short Version for Adults (MHLq-SVa), Connor-Davidson Resilience Scale-10 (CD-RISC-10), The World Health Organization-Five Well-Being Index (WHO-5), Generalized Anxiety Disorder-7 (GAD-7), and Patient Health Questionnaire (PHQ-9) were used to measure the MHL, resilience, well-being and anxiety/depression. Descriptive statistics, Pearson correlation analysis and mediation effect analysis were conducted.

**Results:**

The prevalence of anxiety and depression among nursing students were 39.4% and 9.3% respectively. MHL was negatively correlated with anxiety (r = -0.19, p < 0.001) and depression (r = -0.20, p < 0.001). MHL was positively correlated with resilience (r = 0.43, p < 0.001) and well-being (r = 0.06, p < 0.001). Resilience partially mediated the relationship between MHL and anxiety (indirect effect = -0.040; 95%CI: -0.053 to -0.030), the relationship between MHL and depression (indirect effect = -0.057; 95%CI: -0.073 to -0.040), and the relationship between MHL and well-being (indirect effect = 0.203; 95%CI: 0.177 to 0.228).

**Conclusion:**

Higher MHL levels among nursing students is associated with stronger resilience, further associated with lower levels of anxiety/depression and higher levels of well-being. Our findings provide important guidance for educational administrators and helps them formulate targeted strategies to prevent anxiety/depression among nursing students.

## 1 Introduction

Nursing students, as a special group within the medical education system, carry the core responsibilities of future clinical nursing work (1). During the career growth process, this group needs to simultaneously cope with multiple stressors such as theoretical learning, clinical practice and career development, and these persistent stress factors may trigger significant psychological health risks (2). Of note, the detection rates of anxiety/depression symptoms among nursing students are 54.7% and 28.8% respectively (3), which are significantly higher than general college students (4). The combined effect of multiple pressures such as academic studies and practice has also significantly exacerbated the psychological burden of foreign nursing students, making them face severe mental health challenges (5). The detection rate of anxiety and depression symptoms in this group has remained consistently high, significantly higher than that of the general college student group: for example, in an Australian study, more than 71% of nursing students reported having moderate to extremely high levels of psychological distress (6); another American study also reports the prevalence of depression among nursing students is as high as 37% (7). These mental health issues not only undermine the individual health status of nursing students, but also may reduce the efficiency of future nurse-patient communication and the standardization of nursing operations, thereby potentially exerting negative impacts on the quality of medical services and patient safety (4, 8).

Mental Health Literacy (MHL) has been recognized as a promising intervention target for improving an individual’s mental health (9). Its concept encompasses the knowledge system and cognitive framework regarding the identification, management and prevention of psychological disorders for individuals (10, 11). Moss et al. found that MHL can enhance an individual’s psychological adaptation ability (12), young adults with high levels of MHL also have higher resilience (13). MHL can positively predict higher levels of well-being (14–16). For instance, adolescents and graduate students with high levels of MHL will have higher levels of well-being (12, 14). On the other hand, low levels of MHL are closely associated with psychological issues of medical students (17), and exacerbate the development of emotional problems such as anxiety and depression among college students (18, 19). This association also holds true in a broader student population: lower mental health literacy (such as poor symptom recognition ability and negative attitude towards seeking help) is often accompanied by significantly higher levels of anxiety, depression and stress symptoms (20, 21). However, the relationship between MHL and the mental health of nursing students and the possible mechanisms have not been fully explored.

Well-being refers to the individual’s perception of satisfaction with life and the balance between positive and negative emotions, which is an emotional state that an individual experiences when they perceive that their needs have been met (22). Well-being is an important indicator reflecting an individual’s quality of life (23), and is closely related to mental health (24). College students with high levels of well-being are more satisfied with life and exhibit lower levels of anxiety (25) and depression (26), while individuals with low levels of well-being have higher levels of negative emotions such as anxiety and depression (27).

Resilience reflects the dynamic adaptation process by which individuals maintain psychological homeostasis in adverse circumstances (28). Studies have shown that cultivating resilience can enhance the overall mental health level of college students and can be regarded as an effective way to prevent the occurrence of mental health disorders such as anxiety and depression among college students (29). The formation of good psychological resilience can reduce the occurrence of anxiety/depression among nursing students (30), as well as enhance their levels of well-being (31). Other studies have shown that resilience not only plays a partial mediating role between negative life events and anxiety/depression (30, 32), but also plays a partial mediating role between the mental health and well-being of high school and college students (33, 34). Based on the biopsychosocial model, MHL may have a positive impact on mental health by enhancing psychological resilience through multiple pathways. MHL can firstly promote cognitive remodeling by enhancing an individual’s scientific understanding of mental health (such as understanding the neural mechanism of stress responses), thereby optimizing the functional connection between the prefrontal cortex and the limbic system (35). Secondly, the adaptive coping strategies cultivated by MHL (such as mindfulness techniques) maintain physiological homeostasis by regulating the activity of the HPA axis and immune function (36); furthermore, social resource mobilization under the guidance of MHL (such as active seeking for help) can activate neurosocial mechanisms such as oxytocin secretion and enhance a sense of belonging and security (33). While multiple psychosocial factors (e.g., coping strategies, self-efficacy, social support) may mediate the relationship between MHL and mental health, resilience was prioritized as the focal mediator due to its empirically established role as a meta-construct encompassing adaptive responses to adversity (37). Theoretical models of resilience posit that it integrates cognitive, behavioral, and social resources, aligning with the biopsychosocial lens of this study (38). This parsimonious approach allows for a focused examination of how MHL bolsters overarching psychological resilience, which in turn mitigates mental health risks.

Nursing students in Chinese vocational colleges are prone to mental health problems due to academic pressure (39), pressure of clinical role transition (40), and requirements of clinical operation assessment (41). Although existing studies have initially revealed the relationships between MHL and resilience, anxiety/depression, and well-being, there are still research gaps regarding the mediating role of resilience between MHL and anxiety, depression, and well-being among vocational nursing students. Based on the abovementioned theoretical framework, we propose the following hypotheses:

1. MHL exhibits a significant negative association with symptoms of anxiety and depression.
2. MHL demonstrates a positive correlation with psychological resilience and subjective well-being.
3. Resilience serves as a mediating factor in the relationship between MHL and (a) anxiety, (b) depression, and (c) well-being.

The empirical validation of these hypotheses will contribute to the theoretical foundation for developing evidence-based psychological interventions aimed at mitigating mental health challenges among nursing students.

## 2 Methods

### 2.1 Study design

This study adopted a cross-sectional research design to explore the influence of MHL on the anxiety, depression and well-being among nursing students. The study also examined the mediating role of resilience in the associations between MHL and anxiety, depression, and well-being.

### 2.2 Participants and procedures

This research was approved by the Ethics Review Committee of Henan Vocational College of Nursing. All participants were assured of strict anonymity, with identifiers removed during data aggregation. To safeguard psychological safety, respondents were provided with contact details for local mental health services upon survey completion, alongside a debriefing statement affirming their right to withdraw. The data collection was conducted from October to December 2024 at Henan Vocational College of Nursing. Through convenience sampling, a digital data collection system was constructed through an online survey platform “Questionnaire Star”. The research subjects covered every nursing student at school (including students in the clinical internship stage). Before filling out the questionnaire, all participants were informed of the purpose of the survey. Once participants agreed and scanned the survey QR code, they could enter the filling page and answer the questions. To prevent participants from filling out the questionnaire repeatedly and missing questions, the same participant could only be filled out once by restricting their IP address and mobile phone number. Each question was set as mandatory. In total, the number of nursing students was 1988 at our school, and this survey were completed by 1700 nursing students, among which 1662 valid questionnaires were obtained, since we excluded those providing wrong age (<17 or >25 years old, N=38), and the effective response rate of the questionnaire was 97.8%.

### 2.3 Inclusion and exclusion criteria

This study has formulated clear inclusion and exclusion criteria to ensure the scientific rigor and ethical compliance of the research. Inclusion criteria: participants must be registered students majoring in nursing, who are currently enrolled in college and have voluntarily signed the informed consent form after fully understanding the purpose and process of the study. Exclusion criteria: individuals who are unable to understand the content of the study or unable to effectively express their own situation due to health reasons (A positive answer to “Recently, I have to undergo surgery or have been hospitalized due to illness and have taken sick leave”).

### 2.4 Sample size calculating

The Power Analysis and Sample Size Software (PASS) 2021 (NCSS, LCC. Kaysville, Utah, USA) was used for the estimation of sample size in our study (Formula N =[Z_1-α/2σ_/δ]^2^). The average score and standard deviation (σ) of depression symptoms among nursing students were 8.16 (5.46) (30). The tolerable error (δ) in this study was 8.16 × 1.5%, thus the minimum sample size requirement for nursing students in a two-sided 95% confidence interval (Z=1.96) was 986. However, considering a potential dropout rate of 10%, at least 1096 nursing students were needed to ensure an adequate sample size. Therefore, the final sample size of this study was 1662, which was sufficient according to the previous hypothesis calculation.

## 3 Measurements

### 3.1 General information

General information included the participants’ gender, age, grade (freshmen: the first year; sophomore: the second year; junior: the third year of school), whether they hold the position of class cadre, whether they like the major of nursing, whether they engage in nursing work after graduation, whether they come from single-parent families, the average monthly income per capita of their families, and the educational attainment of their parents.

### 3.2 Mental health literacy

This study adopted the Chinese version of the Mental Health Literacy Questionnaire-Short Version for Adults (MHLq-SVa) developed by Campos et al. (9), and translated by Su et al. (42). The translated Chinese version of the MHLq-SVa has demonstrated good reliability and validity in the college student population (42). It is divided into four dimensions (1): knowledge of mental health problems (e.g., “A person with depression feels very miserable.”; “People with schizophrenia usually have delusions.”) (2), erroneous beliefs/stereotypes (e.g., “Mental disorders don’t affect people’s behaviors.”; “People with mental disorders belong to low-income countries.”, (3) help-seeking and first aid skills (e.g., “If I had a mental disorder, I would seek my relatives’ help.”; “If someone close to me had a mental disorder, I would encourage her/him to look for a psychologist.”, and (4) self-help strategies (e.g., “Physical exercise contributes to good mental health.”; “Sleeping well contributes to good mental health.”). The scale uses the Likert 5-point scoring method (1 = “completely disagree” to 5 = “completely agree”), and the higher the total score, the higher the level of individual mental health literacy (43). The Cronbach’s α of the total scale in this study was 0.89.

### 3.3 Anxiety

The Generalized Anxiety Disorder-7 (GAD-7) scale was developed by Spitzer et al. (44), and is used for screening generalized anxiety and assessing the severity of symptoms. It was adapted into Chinese by He et al. (45), and has been proven to have good reliability and validity in multiple fields (46, 47), and is applicable for screening anxiety among college students (48, 49). The scale consists of 7 items. It is scored on a 4-point scale, with higher scores indicating higher levels of anxiety. A score of 5 or above indicates the presence of anxiety (50). In this study, the Cronbach’s alpha coefficient of GAD-7 was 0.95, demonstrating good internal consistency.

### 3.4 Depression

The Patient Health Questionnaire-9 (PHQ-9) was developed by Koenig et al. (51) to measure an individual’s depressive symptoms. The Chinese version of the PHQ-9 scale was revised by Wang et al. (52) and showed good reliability and validity in the assessment of depression in the general Chinese population. PHQ-9 consists of 9 items and is used to assess the frequency of depressive symptoms experienced by the subjects in the past two weeks. Each depressive symptom is scored from 0 (“not at all”) to 3 (“every day”). Then, the scores of all these questions are summed up. A score of 10 or above is defined as the presence of depressive symptoms (53), and it has been widely used for self-assessment of depression among college students (54, 55). In this study, the Cronbach’s alpha coefficient of PHQ-9 was 0.95, indicating good internal consistency.

### 3.5 Resilience

The Connor-Davidson Resilience Scale-10 (CD-RISC-10) was developed by Connor et al. (56), and translated into Chinese by Yu et al. (57). The scale consists of 10 items, with the score ranging from 1 (representing “never”) to 4 (representing “always”) for each item. The higher the score, the stronger the resilience. The scale has good reliability and validity and can be used as a detection tool for the resilience level of nursing students (30, 58). In this study, the Cronbach’s alpha of the scale was 0.98, indicating good internal consistency.

### 3.6 Well-Being

The World Health Organization-Five Well-Being Index (WHO-5) was developed by the World Health Organization (WHO), aiming to rapidly assess an individual’s subjective mental health and well-being (59). It consists of five simple questions to assess emotional states (such as positive emotions, energy levels, etc.) over the past two weeks (60). The WHO-5 showed good reliability and validity in Chinese population (61). WHO-5 consists of 5 items, each item is scored on a 6-point scale from 0 to 5. The original score is the sum of the 5 items, and the total score ranges from 0 to 25. The higher the total score, the higher the level of well-being (62). In this study, Cronbach’s alpha for WHO-5 was 0.98.

## 4 Data analysis

Firstly, descriptive statistics were conducted on the basic information and measurements of the overall population. Categorical variables were described by counts and percentages, while numerical variables were described by mean ± standard deviation for they passed the normality test (Shapiro-Wilk test), and by median [interquartile range] for those that did not. Then, we grouped the subjects based on the presence or absence of anxiety/depression and compared the differences in basic information and other measurement characteristics between the anxiety group and the non-anxiety group, as well as between the depression group and the non-depression group. Pearson correlation analysis was used to explore the pairwise relationships between mental health literacy level, resilience, and anxiety/depression. Finally, mediation analysis was employed to explore the mediating effects of resilience in the relationship between mental health literacy and anxiety, depression and well-being. We fitted models with anxiety, depression and well-being as dependent variables respectively, controlled basic information as confounding factors, and calculated total, direct, and indirect effects and mediated percentages. Bootstrap method with 5000 resamples was used to fit 95% confidence intervals. In addition, we used Structural Equation Modeling (SEM) to validate our mediating effects and all assessments were fit in one model with mental health literacy as the independent variable, resilience as the mediator, and anxiety/depression/well-being as the outcomes. Before conducting Pearson association analysis and mediation analysis, we performed multicollinearity checks using Variance Inflation Factor (VIF) and the results were acceptable due to VIF < 5 (63). All statistical analyses were conducted using R (4.4.1), and a two-sided p value < 0.05 was considered statistically significant. The R package “mediation” was used to complete the mediation analysis, in accordance with previous studies (64, 65), R package “lavaan” was used to conduct SEM analysis.

## 5 Results

### 5.1 Demographic data of the respondents

Among the 1662 participants, the nursing students were aged between 17 and 25 years old, with an average age of 19.1 years (SD = 1.1). The majority were female, with 1387 participants (83.5%), 805 freshmen (48.4%), 579 sophomores (34.8%), and 278 juniors (16.7%). The total score of the MHL was 63.6 ± 9.2, the total score of the anxiety was 3.8 ± 4.2, the total score of the depression was 4.6 ± 5.2, the total score of the resilience was 34.2 ± 9.2, and the total score of the well-being was 14.7 ± 7.4. Detailed data of the scores are shown in **Table 1**.

**Table 1:** Characteristics of the participants. (N=1662).

Variables	N (%) or mean (SD)
Age	19.1 (1.1)
Gender	
Male	275 (16.5)
Female	1387 (83.5)
Grade	
Freshman	805 (48.4)
Sophomore	579 (34.8)
Junior	278 (16.7)
Romantic relationship	
Yes	303 (18.2)
No	1359(81.8)
Class leaders	
Yes	320 (19.3)
No	1342 (80.7)
Like nursing major	
Yes	1200 (72.2)
No	462 (27.8)
Plan to work as a nurse after graduation	
Yes	671 (40.4)
No	55 (3.3)
Uncertain	936 (56.3)
Left behind	
Yes	442 (26.6)
No	1220 (73.4)
Single parent	
Yes	126 (7.6)
No	1536 (92.4)
Monthly household income (CNY per capita)	
<2000	458 (27.6)
2001-3000	573 (34.5)
3001-4000	333 (20.0)
>4000	298 (17.9)
Father education	
Below junior high school	381 (22.9)
Junior high school	741 (44.6)
Senior high school/technical secondary school	396 (23.8)
Above senior high school	144 (8.7)
Mother education	
Below junior high school	503 (30.3)
Junior high school	685 (41.2)
Senior high school/technical secondary school	340 (20.5)
Above senior high school	134 (8.0)
Assessments	
MHLq-SVa	63.6 (9.2)
Knowledge of mental health problems	23.1 (4.3)
Erroneous beliefs/stereotypes	11.1 (3.1)
Help-seeking and first aid skills	12.2 (2.4)
Self-help strategies	17.1 (4.3)
GAD-7	3.8 (4.2)
PHQ-9	4.6 (5.2)
CD-RISC-10	34.2 (9.2)
WHO-5	14.7 (7.4)

MHLq-SVa, Mental Health Literacy Questionnaire-Short Version for Adults; GAD-7, Generalized Anxiety Disorder-7; PHQ-9, Patient Health Questionnaire-9; CD-RISC-10, Connor-Davidson Resilience Scale-10; WHO-5, The World Health Organization-Five Well-Being Index.

### 5.2 Comparison between the anxiety group and the non-anxiety group

Among all participants, 655 individuals (39.4%) showed anxiety symptoms. The mean age (SD) was 19.1(1.1) years old, 530 (80.9%) are females. The mean scores (SD) of MHL, resilience and well-being in the anxiety group were 60.8 ± 9.0, 30.5 ± 7.9 and 11.7 ± 6.2 respectively. The anxiety group scored significantly lower than the non-anxiety group in terms of MHL (t=-10.03, p < 0.001), resilience (t=-14.42, p < 0.001), and well-being (t=-14.78, p < 0.001), the effect sizes of the difference were reported in **Supplementary Table S1**. Moreover, the anxiety group presented significant group difference with the non-anxiety group in terms of gender (χ² = 4.74, p = 0.029), like nursing major (χ² = 35.83, p < 0.001), and left behind (χ² = 17.96, p < 0.001). Additionally, there were statistically significant differences between the two groups in whether they plan to work as a nurse after graduation (χ² = 12.67, p = 0.002) and the education of their parents (father: χ² = 13.63, p = 0.003; mother: χ² = 8.23, p = 0.042). See **Table 2**.

**Table 2:** Comparisons between nursing students with and without anxiety or depression. (N=1662).

Variables	Anxiety	Non-anxiety	χ^2/^t	p	Depression	Non-depression	χ^2/^t	p
	(N=655, 39.4%)	(N=1007, 60.6%)			(N=154, 9.3%)	(N=1508, 90.7%)		
Age	19.1 (1.1)	19.1 (1.0)	0.5	0.621	19.2 (1.4)	19.1 (1.0)	1.37	0.173
Gender			4.74	0.029			7.49	0.006
Male	125 (19.1)	150 (14.9)			38 (24.7)	237 (15.7)		
Female	530 (80.9)	857 (85.1)			116 (75.3)	1271 (84.3)		
Grade			4.13	0.127			1.19	0.551
Freshman	333 (50.8)	472 (46.9)			77 (50.0)	728 (48.3)		
Sophomore	209 (31.9)	370 (36.7)			48 (31.2)	531 (35.2)		
Junior	113 (17.3)	165 (16.4)			29 (18.8)	249 (16.5)		
Romantic relationship			2.08	0.15			1.98	0.159
Yes	131 (20.0)	172 (17.1)			35 (22.7)	268 (17.8)		
No	524 (80.0)	835 (82.9)			1190 (77.3)	1240 (82.2)		
Class leaders			1.43	0.232			0.68	0.409
Yes	136 (20.8)	184 (18.3)			34 (22.1)	386 (19.0)		
No	519 (79.2)	823 (81.7)			120 (77.9)	1222 (81.0)		
Like nursing major			35.83	<0.001			31.44	<0.001
Yes	419 (64.0)	781 (77.6)			81 (52.6)	1119 (74.2)		
No	236 (36.0)	226 (22.4)			73 (47.4)	389 (25.8)		
Plan to work as a nurse after graduation			12.67	0.002			7.00	0.03
Yes	232 (35.4)	439 (43.6)			48 (31.2)	623 (41.3)		
No	28 (4.3)	27 (2.7)			8 (5.2)	47 (3.1)		
Uncertain	395 (60.3)	541 (53.7)			98 (63.6)	838 (55.6)		
Left behind			17.96	<0.001			2.68	0.102
Yes	212 (32.4)	230 (22.8)			50 (32.5)	392 (26.0)		
No	443 (67.6)	777 (77.2)			104 (67.5)	1116 (74.0)		
Single parent			0.29	0.589			0.07	0.792
Yes	53 (8.1)	73 (7.2)			13 (8.4)	113 (7.5)		
No	602 (91.9)	934 (92.8)			141 (91.6)	1395 (92.5)		
Monthly household income (CNY per capita)			1.76	0.625			7.44	0.059
<2000	191 (29.2)	267 (26.2)			45 (29.2)	413 (27.4)		
2001-3000	225 (34.4)	348 (34.6)			55 (35.7)	518 (34.4)		
3001-4000	124 (18.9)	209 (20.8)			3 (1.9)	314 (20.8)		
>4000	115 (17.6)	183 (18.2)			35 (22.7)	263 (17.4)		
Father education			13.63	0.003			8.46	0.037
Below junior high school	181 (27.6)	200 (19.9)			45 (29.2)	336 (22.3)		
Junior high school	272 (41.5)	469 (46.6)			42 (27.3)	689 (45.7)		
Senior high school/technical secondary school	148 (22.60	248 (24.6)			52 (33.8)	354 (23.5)		
Above senior high school	54 (8.2)	90 (8.9)			15 (9.7)	129 (8.6)		
Mother education			8.23	0.042			8.36	0.039
Below junior high school	215 (32.9)	288 (28.6)			56 (36.4)	447 (29.7)		
Junior high school	242 (36.9)	443 (44.0)			47 (30.5)	638 (42.3)		
Senior high school/technical secondary school	142 (21.7)	198 (19.7)			35 (22.7)	305 (20.2)		
Above senior high school	56 (8.6)	78 (7.7)			16 (10.4)	118 (7.8)		
Assessments								
MHLq-SVa	60.8 (9.0)	65.4 (8.9)	-10.03	<0.001	61.5 (8.8)	63.8 (9.2)	-3.09	0.002
Knowledge of mental health problems	22.6 (4.1)	23.5 (4.3)	-4.3	<0.001	23.5 (4.5)	23.1(4.2)	1.09	0.276
Erroneous beliefs/stereotypes	10.6 (3.1)	11.6 (3.0)	-6.38	<0.001	10.4 (3.7)	11.3 (3.0)	-2.99	0.003
Help-seeking and first aid skills	11.4 (2.3)	12.7 (2.3)	-11.32	<0.001	11.2 (2.6)	12.3 (2.3)	-5.31	<0.001
Self-help strategies	16.3 (2.8)	17.6 (2.8)	-9.47	<0.001	16.5 (2.9)	17.1 (2.9)	-2.76	0.006
GAD-7	8.1 (3.6)	3.0 (3.1)	47.86	<0.001	11.8 (5.5)	3.0 (3.1)	19.67	<0.001
PHQ-9	9.2 (5.1)	3.4 (3.5)	35.86	<0.001	16.2 (5.2)	3.4 (3.5)	29.61	<0.001
CD-RISC-10	30.5 (7.9)	36.6 (9.2)	-14.42	<0.001	31.7 (9.2)	34.5 (9.1)	-3.51	<0.001
WHO-5	11.7 (6.2)	16.7 (7.5)	-14.78	<0.001	11.4 (6.9)	15.0 (7.4)	-6.28	<0.001

### 5.3 Comparison between the depression group and the non-depression group

There were 154 individuals (9.3%) in the depression group, with mean age (SD) was 19.2 (1.4) years, 116 (75.3%) are females. The mean scores (SD) for MHL, resilience, and well-being in the depression group were 61.5 ± 8.8, 31.7 ± 9.2, and 11.4 ± 6.9, respectively. The depression group scored significantly lower than the non-depression group in terms of MHL (t=-3.09, p < 0.001), resilience (t=-3.51, p < 0.001), and well-being (t=-6.28, p < 0.001), the effect sizes of the difference were reported in **Supplementary Table S2**. The depression group presented significant group difference in terms of gender (χ² = 7.49, p = 0.006) and like nursing major (χ² = 31.44, p < 0.001). Additionally, there were statistically significant differences between the two groups in whether plan to work as a nurse after graduation (χ² = 7.00, p = 0.030) and the education of parents (father: χ² = 8.46, p = 0.037; mother: χ² = 8.36, p = 0.039). See **Table 2**.

### 5.4 Correlations between assessments

The study found that MHL was negatively correlated with anxiety symptoms (r = -0.19, p < 0.001) and depression symptoms (r = -0.20, p < 0.001), while demonstrating significant positive correlations with resilience (r = 0.43, p < 0.001) and the well-being (r = 0.33, p < 0.001). Resilience similarly showed negative associations with anxiety symptoms (r = -0.25, p < 0.001) and depression symptoms (r = -0.28, p < 0.001), and was strongly positively correlated with the well-being (r = 0.62, p < 0.001). Both anxiety symptoms (r = -0.26, p < 0.001) and depression symptoms (r = -0.30, p < 0.001) exhibited negative correlations with the well-being (**Table 3**).

**Table 3:** Correlations between assessments (N = 1662).

Variables	MHLq-SVa	WHO-5	CD-RISC-10	GAD-7	PHQ-9
MHLq-SVa	1.00	0.33	0.43	-0.19	-0.20
WHO-5	0.33	1.00	0.62	-0.26	-0.30
CD-RISC-10	0.43	0.62	1.00	-0.25	-0.28
GAD-7	-0.19	-0.26	-0.25	1.00	0.89
PHQ-9	-0.20	-0.30	-0.28	0.89	1.00

all P-value <0.001.

### 5.5 Mediation analysis

**Figure 1:**
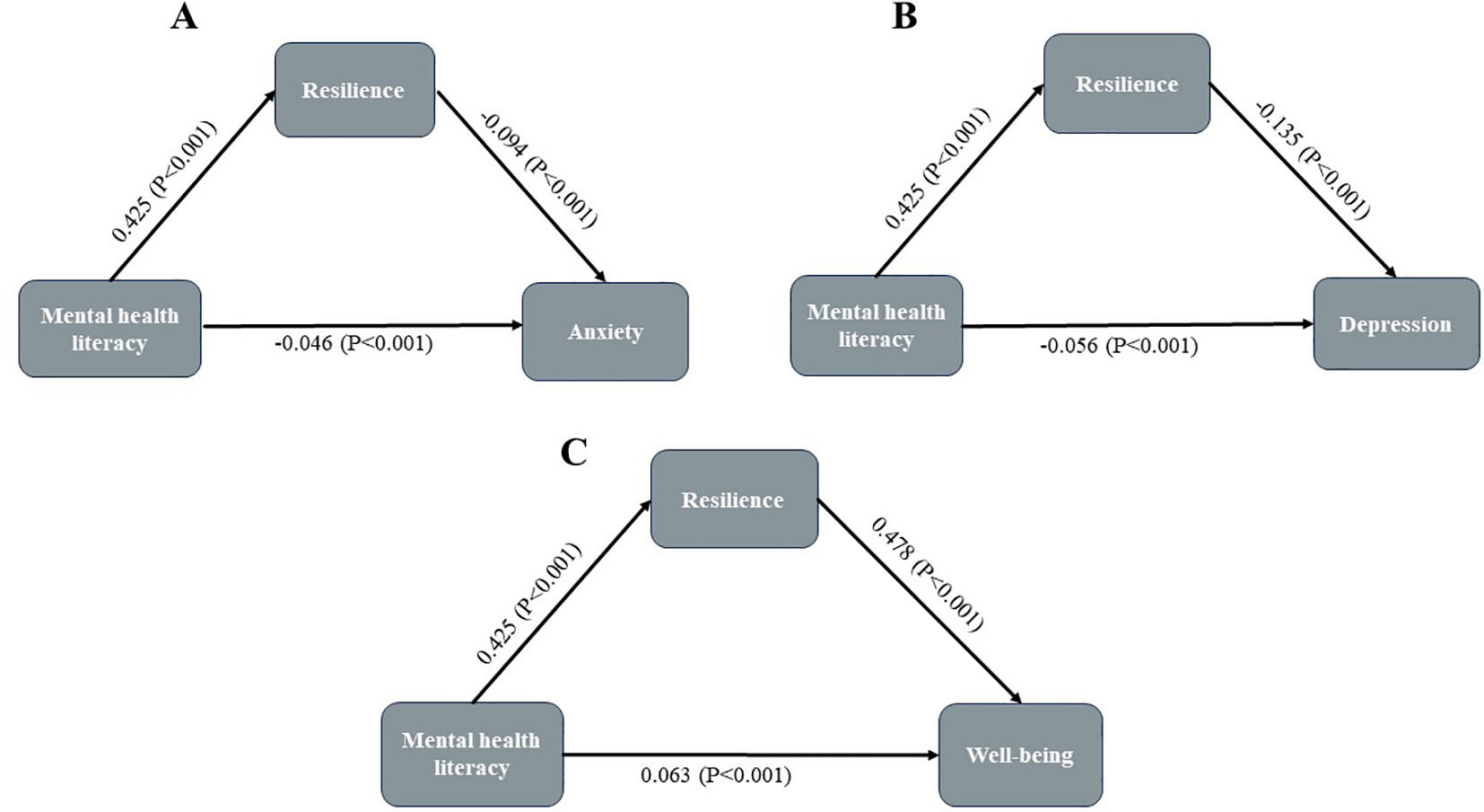
Mediating roles of resilience in the relationship between MHL and anxiety, depression, and well-being. **(A)** MHL -> Resilience -> Anxiety; **(B)** MHL -> Resilience -> Depression; **(C)** MHL -> Resilience -> Well-being.

After controlling for confounding factors with significance in group comparison (gender, like nursing major, plan to work as a nurse after graduation, left behind, parents’ educational attainment), resilience (indirect effect, -0.040; 95% CI, -0.053 to -0.030) partially mediated the relationship between MHL and anxiety (**Figure 1A**), the indirect effect accounting for 46.5% of the total effect. Moreover, resilience (indirect effect, -0.057; 95% CI, -0.073 to -0.040) partially mediated the relationship between MHL and depression (**Figure 1B**), the indirect effect accounting for 50.4% of the total effect. Meanwhile, resilience (indirect effect, 0.203; 95% CI, 0.177 to 0.228) partially mediated the relationship between MHL and well-being (**Figure 1C**), the indirect effect accounting for 76.3% of the total effect. Model summaries were shown in **Table 4**. As shown in **Supplementary Figure 1**, SEM further validated our mediating results, model fit indices were: RMSEA=0.073, SRMR=0.065, CFI=0.891, TLI=0.885.

**Table 4:** Mediation model summaries.

Coefficients	Estimate	95% CI		P-value
		Lower	Upper	
Path: MHL -> Resilience -> Anxiety				
Indirect effect	-0.040	-0.053	-0.030	<0.001
Direct effect	-0.046	-0.075	-0.019	<0.001
Total effect	-0.086	-0.111	-0.061	<0.001
Mediated proportions	0.465	0.301	0.695	<0.001
Path: MHL -> Resilience -> Depression				
Indirect effect	-0.057	-0.073	-0.040	<0.001
Direct effect	-0.056	-0.086	-0.024	<0.001
Total effect	-0.113	-0.144	-0.076	<0.001
Mediated proportions	0.504	0.345	0.729	<0.001
Path: MHL -> Resilience -> Well-being				
Indirect effect	0.203	0.177	0.228	<0.001
Direct effect	0.063	0.028	0.088	<0.001
Total effect	0.266	0.230	0.303	<0.001
Mediated proportions	0.763	0.666	0.877	<0.001

## 6 Discussion

This study is the first to explore the relationships between MHL and resilience, anxiety, depression and well-being among nursing students in China. It was found that the higher the levels of MHL, resilience and well-being, the lower the anxiety and depression of the nursing students. Meanwhile, resilience can respectively mediate the influence of MHL on anxiety, depression and well-being. Overall, the results of this study verified the proposed hypotheses.

### 6.1 The prevalence of anxiety/depression among nursing students

The results of this study showed that the anxiety and depression scores of vocational nursing students were 3.8 ± 4.2 and 4.6 ± 5.2, respectively, which were lower than the results of Ye et al. and Shi et al. on undergraduate nursing students (30, 54). This difference may be attributed to multiple factors such as the difference between academic pressure and educational goals, the uncertainty of career development expectations, the pressure of social and family expectations, the perfection of psychological support system, and the particularity of age and life stage. In addition, the detection rates of anxiety and depression in this study were 39.3% and 9.3%, respectively, among which the detection rate of anxiety was higher than that of Gong and Ribeiro et al, while the detection rate of depression was lower than that of Gong and Ribeiro et al (3, 66). This discrepancy may be attributed to multiple factors such as screening criteria of subjects, differences in measurement tools, and heterogeneity in research methods. It is also possible that, compared with undergraduate nursing students, vocational nursing students face relatively lower academic pressure (as their training objectives focus more on practical skills rather than academic research), have clearer career development expectations (mainly by directly entering clinical nursing positions), and bear less pressure from social and family achievement expectations. These factors work together to make the detection rate of depression relatively low.

### 6.2 The association between MHL and anxiety, depression, and well-being

This study is the first to reveal that among vocational college nursing students, MHL is negatively correlated with anxiety/depression, which is consistent with the previous research results on Chinese college students and adults (67, 68). MHL is an important resource for mental health (69), and it can effectively improve an individual’s psychological adaptation ability (12). Individuals with high MHL can identify mental health problems at an early stage, better understand the causes or triggers of psychological problems or diseases, know how to deal with them, and have a less stigmatizing attitude towards psychological problems or diseases (70), thus being able to timely apply techniques or methods for self-adjustment or seeking help, thereby reducing the degree of negative emotions such as anxiety/depression (71).

Furthermore, among the nursing students in vocational colleges, MHL is positively correlated with well-being, which is consistent with the previous findings on adolescents and graduate students (12, 14). Well-being is one of the important indicators reflecting mental health, representing the comprehensive state of an individual’s psychological experience. Individuals with higher MHL can better recognize, express, and regulate their emotions, thereby more effectively coping with challenges and pressures in life, maintaining a positive mindset, and adopting positive behavioral strategies to enhance their well-being (15).

### 6.3 The mediating role of resilience between MHL and anxiety, depression and well-being

A major finding of this study is that resilience plays a partial mediating role between MHL and anxiety/depression. MHL is not only directly associated with the anxiety/depression levels of nursing students, but also indirectly associated with their anxiety/depression levels through the mediating of resilience. The mediating effect proportions are 46.5% and 50.46% respectively.

College students with higher MHL levels can actively cope with stress and adversity in study and life (72), and resilience reflects the dynamic adaptation process of an individual to maintain psychological stability in adversity (28). Therefore, individuals with higher MHL levels have stronger resilience. MHL enhances an individual’s adaptability in adversity through mechanisms such as cognitive reconstruction (such as rational analysis of stressors), resource mobilization (actively seeking social support or professional assistance), emotion regulation (managing negative emotions through techniques like mindfulness), and self-efficacy improvement (boosting confidence in coping with challenges), thereby improving psychological resilience (43, 73). Compared with students from other majors, nursing students are more likely to encounter adverse life events, including academic pressure and death in the working environment. These negative events are key factors increasing the susceptibility to anxiety, depression and other mental health problems (30), and resilience as a key protective factor can help individuals effectively cope with negative emotions such as anxiety and depression (74). From this, we can infer that nursing students with higher MHL levels have stronger resilience, and the stronger their individual anti-stress ability is, the lower the incidence of anxiety and depression will be. Therefore, nursing students with good MHL show lower anxiety/depression levels under higher level of resilience. Future research can enhance the MHL of nursing students and strengthen their resilience, effectively lowering the occurrence of mental health problems such as anxiety and depression in nursing students, and ensure the infusion of new forces in the future medical industry.

Another important finding of this study is that resilience also plays a partial mediating role between MHL and well-being. MHL not only directly correlates the well-being levels of nursing students but also indirectly correlates with well-being through mediating role of resilience. Of note, the mediating proportions are 76.3% of the total effect of MHL on well-being. Resilience refers to an effective coping mechanism that individuals adopt when confronted with stressful situations (such as loss, difficulties, etc.), enabling them to achieve good adaptive outcomes (75). Individuals with high levels of resilience possess strong psychological recovery capabilities and can adapt well in the face of adversity. These individuals often present higher levels of well-being (31). The relationship between MHL and well-being is largely mediated by psychological resilience, indicating that in practice, focusing on an individual’s MHL and psychological resilience levels may be more conducive to enhancing well-being (12, 76). Therefore, it can be inferred that nursing students with higher levels of MHL tend to have stronger well-being. Thus, students with good MHL can enhance their well-being through resilience. This finding is helpful for us to better understand how MHL affects the well-being of nursing students and provides some effective intervention strategies.

This study represents the pioneering effort to systematically investigate the relationship between MHL, psychological resilience, anxiety and depression, as well as well-being among Chinese nursing students. The findings confirm that MHL contributes to improving mental health by enhancing psychological resilience. Specifically, the research demonstrates that MHL not only directly influences the levels of anxiety and depression and well-being in nursing students but also exerts a protective effect by reinforcing psychological resilience as a critical mediating variable. Mechanisms such as cognitive restructuring (e.g., rational analysis of stressors), resource mobilization (e.g., active seeking of social support), and emotion regulation (e.g., mindfulness training) (43, 77) significantly enhance the psychological adaptability of nursing students in response to academic pressure and clinical environment challenges (78). These insights offer valuable implications for mental health interventions in nursing education. Future research could build upon these findings to design targeted MHL intervention programs, providing both theoretical foundations and practical guidance for fostering nursing talents with robust psychological qualities.

## 7 Conclusions

This study found that among nursing students, higher mental health literacy (MHL) was associated with greater resilience and well-being, and lower anxiety/depression. Resilience partially mediated the effects of MHL on anxiety, depression, and well-being. These findings highlight the importance of fostering MHL and resilience in mental health interventions for nursing students. Future studies using longitudinal designs to further validated our findings are needed.

## 8 Limitations

Although this study has made valuable findings in exploring the influence of MHL on the resilience, and anxiety/depression/well-being of vocational college nursing students, it has provided a new perspective for the existing knowledge and information on improving the anxiety/depression/well-being of nursing students. However, there are still certain limitations. Firstly, this study adopts a cross-sectional design, which cannot establish the causal relationship between the research variables. Therefore, future research should consider adopting a longitudinal design to overcome this limitation and more clearly reveal the dynamic relationship between the variables. The assessment tools are in the form of self-reports and may have self-report bias or social desirability bias. In the future, more objective assessments, such as face-to-face interviews, can be adopted. Also, we didn’t consider the relationship between lecturer and student interactions, academic stress, and clinical exposure as covariates, which are important factors associated with nursing students’ mental health (79, 80). Secondly, the convenience sampling method can lead to sampling errors and reduce the representativeness of the samples. In subsequent studies, it is recommended to adopt the random sampling method. Moreover, the participants in this study mainly come from nursing students of a vocational college in Henan Province in the central-eastern part of China. They do not reflect nursing students from other regions of China, which may limit the representativeness of the sample. The study’s regional focus on Henan Province necessitates caution in extrapolating results. Henan’s socioeconomic and healthcare landscape—such as urban-rural disparities in education access—may uniquely shape MHL adoption and resilience pathways. Cross-province comparisons in China, where policies and mental health infrastructure vary, are needed to assess broader applicability. Similarly, international generalizations require validation in cultures with distinct mental health literacy frameworks (e.g., individualistic vs. collectivist societies). Future studies are warranted to cover a much more representative sample of nursing students to verify our findings. Moreover, the gender distribution of our sample (83% female) may influence the generalizability of findings. While this reflects the demographic profile of the target population (e.g., higher female engagement in mental health surveys), gender differences in resilience mechanisms or MHL uptake remain plausible (81). Future studies should explicitly test gender moderation effects to determine whether the observed relationships hold across subgroups, particularly in male populations where mental health help-seeking behaviors may differ. Finally, there are still other potential factors that may mediate the relationship between MHL and mental health, including self-efficacy (20), social support (77), all of which deserve further exploration.

## Data Availability

The raw data supporting the conclusions of this article will be made available by the authors, without undue reservation.

## References

[1] Ciezar Andersen S Campbell T White D King-Shier K. An intervention to improve mental and physical health of undergraduate nursing students. Can J Nurs Res. (2024) 56:317–28. doi: 10.1177/08445621241248308 PMC11834340 38706094

[2] Amaducci Cde M Mota DD Pimenta CA. Fatigue among nursing undergraduate students. Rev Esc Enferm USP. (2010) 44:1052–8. doi: 10.1590/s0080-62342010000400028 21337789

[3] Gong J Pan Z Liu A Zheng H Jiang Y Zhu G. A typical correlation analysis of anxiety, depression and professional adaptability among 799 undergraduate nursing students. J Nurs. (2020) 27:70–3. doi: 10.16460/j.issn1008-9969.2020.02.070

[4] Aloufi MA Jarden RJ Gerdtz MF Kapp S. Reducing stress, anxiety and depression in undergraduate nursing students: systematic review. Nurse Educ Today. (2021) 102:104877. doi: 10.1016/j.nedt.2021.104877 33905898

[5] Chernomas WM Shapiro C. Stress, depression, and anxiety among undergraduate nursing students. Int J Nurs Educ Scholarsh. (2013) 10. doi: 10.1515/ijnes-2012-0032 24200536

[6] Gibbons C Dempster M Moutray M. Stress and eustress in nursing students. J Adv Nurs. (2008) 61:282–90. doi: 10.1111/j.1365-2648.2007.04497.x 18197862

[7] Tung YJ Lo KKH Ho RCM Tam WSW. Prevalence of depression among nursing students: A systematic review and meta-analysis. Nurse Educ Today. (2018) 63:119–29. doi: 10.1016/j.nedt.2018.01.009 29432998

[8] Al-Mamun F Mamun MA Kaggwa MM Mubarak M Hossain MS MM AL. The prevalence of nomophobia: A systematic review and meta-analysis. Psychiatry Res. (2025) 349:116521. doi: 10.1016/j.psychres.2025.116521 40334351

[9] Campos L Dias P Costa M Rabin L Miles R Lestari S. Mental health literacy questionnaire-short version for adults (Mhlq-Sva): validation study in China, India, Indonesia, Portugal, Thailand, and the United States. BMC Psychiatry. (2022) 22:713. doi: 10.1186/s12888-022-04308-0 36384505 PMC9668212

[10] Jorm AF Korten AE Jacomb PA Christensen H Rodgers B Pollitt P. Mental health literacy”: A survey of the public’s ability to recognise mental disorders and their beliefs about the effectiveness of treatment. Med J Aust. (1997) 166:182–6. doi: 10.5694/j.1326-5377.1997.tb140071.x 9066546

[11] ElKhalil R AlMekkawi M O’Connor M Masuadi E Sherif M Belfakir M. Measurement properties of the mental health literacy scale (Mhls): A systematic review. Asian J Psychiatr. (2024) 101:104214. doi: 10.1016/j.ajp.2024.104214 39255647

[12] Moss RA Gorczynski P Sims-Schouten W Heard-Laureote K Creaton J. Mental health and wellbeing of postgraduate researchers: exploring the relationship between mental health literacy, help-seeking behaviour, psychological distress, and wellbeing. Higher Educ Res Dev. (2021) 41:1168–83. doi: 10.1080/07294360.2021.1906210

[13] Afek A Ben-Avraham R Davidov A Berezin Cohen N Ben Yehuda A Gilboa Y. Psychological Resilience, Mental Health, and Inhibitory Control among Youth and Young Adults under Stress. Front Psychiatry. (2020) 11:608588. doi: 10.3389/fpsyt.2020.608588 33584372 PMC7874000

[14] Bjørnsen HN Espnes GA Eilertsen MB Ringdal R Moksnes UK. The relationship between positive mental health literacy and mental well-being among adolescents: implications for school health services. J Sch Nurs. (2019) 35:107–16. doi: 10.1177/1059840517732125 PMC7323733 28950750

[15] Mahmoodi SMH Rasoulian M Khodadoust E Jabari Z Emami S Ahmadzad-Asl M. The well-being of Iranian adult citizens; is it related to mental health literacy? Front Psychiatry. (2023) 14:1127639. doi: 10.3389/fpsyt.2023.1127639 37215675 PMC10196501

[16] Nalipay MJN Chai CS Jong MS King RB Mordeno IG. Positive mental health literacy for teachers: adaptation and construct validation. Curr Psychol. (2023), 1–11. doi: 10.1007/s12144-023-04694-y PMC10152013 37359647

[17] Elsheshtawy E Simon M Golchinheydari S. Assessment of mental health literacy of depression and suicide among undergraduate medical students: A cross-sectional study. Arab J Psychiatry. (2020) 31:159–68. doi: 10.12816/0056867

[18] Huang X Wang X Hu J Xue Y Wei Y Wan Y. Inadequate mental health literacy and insufficient physical activity potentially increase the risks of anxiety and depressive symptoms in Chinese college students. Front Psychiatry. (2021) 12:753695. doi: 10.3389/fpsyt.2021.753695 34867541 PMC8637166

[19] Zhang X Yue H Hao X Liu X Bao H. Exploring the relationship between mental health literacy and psychological distress in adolescents: A moderated mediation model. Prev Med Rep. (2023) 33:102199. doi: 10.1016/j.pmedr.2023.102199 37223554 PMC10201844

[20] O’Connor M Casey L. The mental health literacy scale (Mhls): A new scale-based measure of mental health literacy. Psychiatry Res. (2015) 229:511–6. doi: 10.1016/j.psychres.2015.05.064 26228163

[21] Gulliver A Griffiths KM Christensen H. Perceived barriers and facilitators to mental health help-seeking in young people: A systematic review. BMC Psychiatry. (2010) 10:113. doi: 10.1186/1471-244x-10-113 21192795 PMC3022639

[22] Lucas RE Diener E Suh E. Discriminant validity of well-being measures. J Pers Soc Psychol. (1996) 71:616–28. doi: 10.1037//0022-3514.71.3.616 8831165

[23] Avedissian T Alayan N. Adolescent well-being: A concept analysis. Int J Ment Health Nurs. (2021) 30:357–67. doi: 10.1111/inm.12833 33394556

[24] Pan J Xu T Li D. The relationship between mental health literacy and social well-being: A longitudinal study in China. Behav Sci (Basel). (2024) 15. doi: 10.3390/bs15010029 PMC11763095 39851833

[25] Dias Lopes LF Chaves BM Fabrício A Porto A MaChado de Almeida D Obregon SL. Analysis of well-being and anxiety among university students. Int J Environ Res Public Health. (2020) 17. doi: 10.3390/ijerph17113874 PMC7312407 32486134

[26] Marshall EJ Brockman RN. The relationships between psychological flexibility, self-compassion, and emotional well-being. J Cognit Psychother. (2016) 30:60–72. doi: 10.1891/0889-8391.30.1.60 32755906

[27] Kinderman P Tai S Pontin E Schwannauer M Jarman I Lisboa P. Causal and mediating factors for anxiety, depression and well-being. Br J Psychiatry. (2015) 206:456–60. doi: 10.1192/bjp.bp.114.147553 25858180

[28] Sisto A Vicinanza F Campanozzi LL Ricci G Tartaglini D Tambone V. Towards a transversal definition of psychological resilience: A literature review. Medicina (Kaunas). (2019) 55. doi: 10.3390/medicina55110745 PMC6915594 31744109

[29] Lau WKW. The role of resilience in depression and anxiety symptoms: A three-wave cross-lagged study. Stress Health. (2022) 38:804–12. doi: 10.1002/smi.3136 35191153

[30] Ye X Yang G Zhang W Toussaint L Zhao F. Relationship of negative life events with depression and anxiety in nursing students: A moderated mediation model of resilience and gender. BMC Nurs. (2025) 24:58. doi: 10.1186/s12912-024-02661-x 39825328 PMC11740486

[31] Almutlaq BA Almohaimeed LA Kahin SA Alsubaie MS. Psychological resilience and well-being among a sample of Saudi. Saudi Med J. (2024) 45:963–7. doi: 10.15537/smj.2024.45.9.20240467 PMC11376708 39218473

[32] Liu WJ Zhou L Wang XQ Yang BX Wang Y Jiang JF. Mediating role of resilience in relationship between negative life events and depression among Chinese adolescents. Arch Psychiatr Nurs. (2019) 33:116–22. doi: 10.1016/j.apnu.2019.10.004 31753216

[33] Davydov DM Stewart R Ritchie K Chaudieu I. Resilience and mental health. Clin Psychol Rev. (2010) 30:479–95. doi: 10.1016/j.cpr.2010.03.003 20395025

[34] Schultze-Lutter F Schimmelmann BG Schmidt SJ. Resilience, risk, mental health and well-being: associations and conceptual differences. Eur Child Adolesc Psychiatry. (2016) 25:459–66. doi: 10.1007/s00787-016-0851-4 27105994

[35] Rutten BP Hammels C Geschwind N Menne-Lothmann C Pishva E Schruers K. Resilience in mental health: linking psychological and neurobiological perspectives. Acta Psychiatr Scand. (2013) 128:3–20. doi: 10.1111/acps.12095 23488807 PMC3746114

[36] Ungar M Theron L. Resilience and mental health: how multisystemic processes contribute to positive outcomes. Lancet Psychiatry. (2020) 7:441–8. doi: 10.1016/s2215-0366(19)30434-1 31806473

[37] Luthar SS Cicchetti D Becker B. The construct of resilience: A critical evaluation and guidelines for future work. Child Dev. (2000) 71:543–62. doi: 10.1111/1467-8624.00164 PMC1885202 10953923

[38] Bennett JM Rohleder N Sturmberg JP. Biopsychosocial approach to understanding resilience: stress habituation and where to intervene. J Eval Clin Pract. (2018) 24:1339–46. doi: 10.1111/jep.13052 30338615

[39] Zhao X Sun J Jia S Chen Y Peng Y. Effectiveness of the three-line relaxation-based group intervention on mental stress management among nursing students. Chin J Sch Health. (2019) 40:1040–2. doi: 10.16835/j.cnki.1000-9817.2019.07.022

[40] Wang C Cheng Y Wu H. Analysis of the clinical practice situation of vocational college nursing students and countermeasures. Educ Vocation. (2012) 20:189–90. doi: 10.13615/j.cnki.1004-3985.2012.20.008

[41] Cui Y Tian S Zhang X Wang L Zhang D. Investigation and analysis on the impact of clinical practice on the psychological stress of nursing students. Higher Med Educ China. (2024) 10:105–6.

[42] Su D Liao J Chen J Peng K. Reliability and validity of mental health literacy questionnaire-short version for adults in Chinese college students. Chin J Clin Psychol. (2024) 06):1357–61. doi: 10.16128/j.cnki.1005-3611.2024.06.030

[43] Jorm AF. Mental health literacy: empowering the community to take action for better mental health. Am Psychol. (2012) 67:231–43. doi: 10.1037/a0025957 22040221

[44] Spitzer RL Kroenke K Williams JB Löwe B. A brief measure for assessing generalized anxiety disorder: the Gad-7. Arch Intern Med. (2006) 166:1092–7. doi: 10.1001/archinte.166.10.1092 16717171

[45] He X Li C Qian J Cui H Wu W. Reliability and validity of a generalized anxiety disorder scale in general hospital outpatients. Shanghai Arch Psychiatry. (2010) 22:200–3.

[46] Bolgeo T Di Matteo R Simonelli N Dal Molin A Lusignani M Bassola B. Psychometric properties and measurement invariance of the 7-item general anxiety disorder scale (Gad-7) in an Italian coronary heart disease population. J Affect Disord. (2023) 334:213–9. doi: 10.1016/j.jad.2023.04.140 37149049

[47] Camargo L Herrera-Pino J Shelach S Soto-Añari M Porto MF Alonso M. Gad-7 generalised anxiety disorder scale in Colombian medical professionals during the Covid-19 pandemic: construct validity and reliability. Rev Colomb Psiquiatr (Engl Ed). (2023) 52:245–50. doi: 10.1016/j.rcpeng.2021.06.011 37863769

[48] Byrd-Bredbenner C Eck K Quick V. Gad-7, Gad-2, and Gad-mini: psychometric properties and norms of university students in the United States. Gen Hosp Psychiatry. (2021) 69:61–6. doi: 10.1016/j.genhosppsych.2021.01.002 33571925

[49] Martínez-Vázquez S Martínez-Galiano JM Peinado-Molina RA Gutiérrez-Sánchez B Hernández-Martínez A. Validation of general anxiety disorder (Gad-7) questionnaire in Spanish nursing students. PeerJ. (2022) 10:e14296. doi: 10.7717/peerj.14296 36340193 PMC9635356

[50] Liu J Liu Z Zhou Y Wu L Wang N Liu X. The relationship between plant-based diet indices and sleep health in older adults: the mediating role of depressive symptoms and anxiety. Nutrients. (2024) 16. doi: 10.3390/nu16193386 PMC11478969 39408353

[51] Bergmans RS Malecki KM. The association of dietary inflammatory potential with depression and mental well-being among U.S. Adults. Prev Med. (2017) 99:313–9. doi: 10.1016/j.ypmed.2017.03.016 PMC5484161 28342730

[52] Wang W Bian Q Zhao Y Li X Wang W Du J. Reliability and validity of the Chinese version of the patient health questionnaire (Phq-9) in the general population. Gen Hosp Psychiatry. (2014) 36:539–44. doi: 10.1016/j.genhosppsych.2014.05.021 25023953

[53] Kroenke K Spitzer RL Williams JB. The Phq-9: validity of a brief depression severity measure. J Gen Intern Med. (2001) 16:606–13. doi: 10.1046/j.1525-1497.2001.016009606.x PMC1495268 11556941

[54] Shi X Wu Y Wang X Xu J Miao J Zang S. Factors associated with nursing students’ Mental health-related stigma: A multisite cross-sectional study. Nurse Educ Today. (2024) 142:106346. doi: 10.1016/j.nedt.2024.106346 39146919

[55] Wickramasinghe A Essén B Surenthirakumaran R Axemo P. Prevalence of Depression among Students at a Sri Lankan University: A Study Using the Patient Health Questionnaire-9 (Phq-9) During the Covid-19 Pandemic. BMC Public Health. (2023) 23:528. doi: 10.1186/s12889-023-15427-y 36941588 PMC10026232

[56] Connor KM Davidson JR. Development of a new resilience scale: the connor-davidson resilience scale (Cd-Risc). Depress Anxiety. (2003) 18:76–82. doi: 10.1002/da.10113 12964174

[57] Yu X-n Lau JTF Mak WWS Zhang J Lui WWS Zhang J. Factor structure and psychometric properties of the Connor-Davidson resilience scale among Chinese adolescents. Compr Psychiatry. (2011) 52:218–24. doi: 10.1016/j.comppsych.2010.05.010 21295229

[58] Ye Z Ruan X Zeng Z Xie Q Cheng M Peng C. Psychometric properties of 10-item Connor-Davidson resilience scale among nursing students. J Nursing(China). (2016) 23:9–13. doi: 10.16460/j.issn1008-9969.2016.21.009

[59] Bech P Gudex C Johansen KS. The who (Ten) well-being index: validation in diabetes. Psychother Psychosom. (1996) 65:183–90. doi: 10.1159/000289073 8843498

[60] Topp CW Østergaard SD Søndergaard S Bech P. The who-5 well-being index: A systematic review of the literature. Psychother Psychosom. (2015) 84:167–76. doi: 10.1159/000376585 25831962

[61] Fung S-f Kong CYW Liu Y-m Huang Q Xiong Z Jiang Z. Validity and psychometric evaluation of the Chinese version of the 5-item who well-being index. Front Public Health. (2022) 10:872436. doi: 10.3389/fpubh.2022.872436 35433612 PMC9005828

[62] Primack BA. The who-5 wellbeing index performed the best in screening for depression in primary care. ACP J Club. (2003) 139:48. doi: 10.7326/ACPJC-2003-139-2-048 12954040

[63] Kim JH. Multicollinearity and misleading statistical results. Korean J Anesthesiol. (2019) 72:558–69. doi: 10.4097/kja.19087 PMC6900425 31304696

[64] Nong Y Wu G Lu J Wei X Yu D. The mediating role of obesity in the development of depression in individuals with diabetes: A population-based study from Nhanes 2005–2014. J Affect Disord. (2024) 351:977–82. doi: 10.1016/j.jad.2024.02.036 38355056

[65] Yin J Gong R Zhang M Ding L Shen T Cai Y. Associations between sleep disturbance, inflammatory markers and depressive symptoms: mediation analyses in a large Nhanes community sample. Prog Neuropsychopharmacol Biol Psychiatry. (2023) 126:110786. doi: 10.1016/j.pnpbp.2023.110786 37178815

[66] Ribeiro A Fernandes MA Vedana KGG Lira JAC Barbosa NS Rocha EP. Mental health and university dropout among nursing students: A cross-sectional study. Nurse Educ Today. (2025) 147:106571. doi: 10.1016/j.nedt.2025.106571 39854879

[67] Yao ZY Wang T Yu YK Li R Sang X Fu YN. Mental health literacy and suicidal ideation among Chinese college students: the mediating role of depressive symptoms and anxiety symptoms. J Affect Disord. (2023) 339:293–301. doi: 10.1016/j.jad.2023.07.050 37437723

[68] Zhong SL Wang SB Ding KR Tan WY Zhou L. Low mental health literacy is associated with depression and anxiety among adults: A population-based survey of 16,715 adults in China. BMC Public Health. (2024) 24:2721. doi: 10.1186/s12889-024-20020-y 39370527 PMC11456243

[69] Tay JL Tay YF Klainin-Yobas P. Mental health literacy levels. Arch Psychiatr Nurs. (2018) 32:757–63. doi: 10.1016/j.apnu.2018.04.007 30201205

[70] Rafal G Gatto A DeBate R. Mental health literacy, stigma, and help-seeking behaviors among male college students. J Am Coll Health. (2018) 66:284–91. doi: 10.1080/07448481.2018.1434780 29419361

[71] Guo S Yang Y Liu F Li F. The awareness rate of mental health knowledge among Chinese adolescent: A systematic review and meta-analysis. Med (Baltimore). (2020) 99:e19148. doi: 10.1097/md.0000000000019148 PMC7035058 32049839

[72] Zhang S Yang R Cui Y Zhou Y Jiang L Xi J. Negative life events, inadequate mental health literacy, and emotional symptoms among Chinese college students: A school-based longitudinal prospective study. Int J Ment Health Syst. (2025) 19:14. doi: 10.1186/s13033-025-00672-y 40307830 PMC12042421

[73] Kutcher S Wei Y Coniglio C. Mental health literacy: past, present, and future. Can J Psychiatry. (2016) 61:154–8. doi: 10.1177/0706743715616609 PMC4813415 27254090

[74] Lyu C Ma R Hager R Porter D. The relationship between resilience, anxiety, and depression in Chinese collegiate athletes. Front Psychol. (2022) 13:921419. doi: 10.3389/fpsyg.2022.921419 36033035 PMC9416885

[75] Imran A Tariq S Kapczinski F de Azevedo Cardoso T. Psychological resilience and mood disorders: A systematic review and meta-analysis. Trends Psychiatry Psychother. (2024) 46:e20220524. doi: 10.47626/2237-6089-2022-0524 36215270 PMC11332678

[76] Luo S Zhou B. A study on the relationship between mental health and subjective well-being of college students with left-behind experience based on the mediating role of resilience. J Southwest JiaoTong (Social Sciences). (2017) 18:72–8.

[77] Wei Y McGrath PJ Hayden J Kutcher S. Mental health literacy measures evaluating knowledge, attitudes and help-seeking: A scoping review. BMC Psychiatry. (2015) 15:291. doi: 10.1186/s12888-015-0681-9 26576680 PMC4650294

[78] O’Connor M Casey L Clough B. Measuring mental health literacy–a review of scale-based measures. J Ment Health. (2014) 23:197–204. doi: 10.3109/09638237.2014.910646 24785120

[79] Karaca A Yildirim N Cangur S Acikgoz F Akkus D. Relationship between mental health of nursing students and coping, self-esteem and social support. Nurse Educ Today. (2019) 76:44–50. doi: 10.1016/j.nedt.2019.01.029 30769177

[80] Hagenauer G Volet SE. Teacher-Student Relationship at University: An Important yet under-Researched Field. Oxf Rev Educ. (2014) 40:370–88. doi: 10.1080/03054985.2014.921613 PMC4864866 27226693

[81] Lee HY Hwang J Ball JG Lee J Yu Y Albright DL. Mental health literacy affects mental health attitude: is there a gender difference? Am J Health Behav. (2020) 44:282–91. doi: 10.5993/ajhb.44.3.1 32295676

