# Supplementary material for: The impact of mental health literacy on depression, anxiety and well-being among vocational nursing students: mediating roles of resilience
Source: Front Psychiatry. 2025 Jun 30;16:1585642. doi: 10.3389/fpsyt.2025.1585642 (PMC12256488; doi:10.3389/fpsyt.2025.1585642)
Supplement: Supplementary file 1 [file Supplementaryfile1.docx]

| Table S1 Effect sizes in the comparisons between anxiety and non-anxiety of all assessments. | | | | |
| --- | --- | --- | --- | --- |
| **Variables** | **Anxiety**  **(N=655, 39.4%)** | **Non-anxiety**  **(N=1007, 60.6%)** | **Cohen's d** | |
|  |  |  |  |  |
| MHLq-SVa | 60.8 (9.0) | 65.4 (8.9) | 0.504 | medium |
| Knowledge of mental health problems | 22.6 (4.1) | 23.5 (4.3) | 0.214 | small |
| Erroneous beliefs/stereotypes | 10.6 (3.1) | 11.6 (3.0) | 0.322 | small |
| Help-seeking and first aid skills | 11.4 (2.3) | 12.7 (2.3) | 0.566 | medium |
| Self-help strategies | 16.3 (2.8) | 17.6 (2.8) | 0.476 | small |
| GAD-7 | 8.1 (3.6) | 3.0 (3.1) | -2.807 | large |
| PHQ-9 | 9.2 (5.1) | 3.4 (3.5) | -2.059 | large |
| CD-RISC-10 | 30.5 (7.9) | 36.6 (9.2) | 0.701 | medium |
| WHO-5 | 11.7 (6.2) | 16.7 (7.5) | 0.713 | medium |

| Table S2 Effect sizes in the comparisons between depression and non-anxiety of all assessments. | | | | |
| --- | --- | --- | --- | --- |
| **Variables** | **Depression**  **(N=154, 9.3%)** | **Non-Depression**  **(N=1508, 90.7%)** | **Cohen's d** | |
|  |  |  |  | |
| MHLq-SVa | 61.5 (8.8) | 63.8 (9.2) | 0.251 | small |
| Knowledge of mental health problems | 23.5 (4.5) | 23.1(4.2) | -0.096 | negligible |
| Erroneous beliefs/stereotypes | 10.4 (3.7) | 11.3 (3.0) | 0.296 | small |
| Help-seeking and first aid skills | 11.2 (2.6) | 12.3 (2.3) | 0.486 | small |
| Self-help strategies | 16.5 (2.9) | 17.1 (2.9) | 0.231 | small |
| GAD-7 | 11.8 (5.5) | 3.0 (3.1) | -2.607 | large |
| PHQ-9 | 16.2 (5.2) | 3.4 (3.5) | -3.460 | large |
| CD-RISC-10 | 31.7 (9.2) | 34.5 (9.1) | 0.299 | small |
| WHO-5 | 11.4 (6.9) | 15.0 (7.4) | 0.500 | medium |


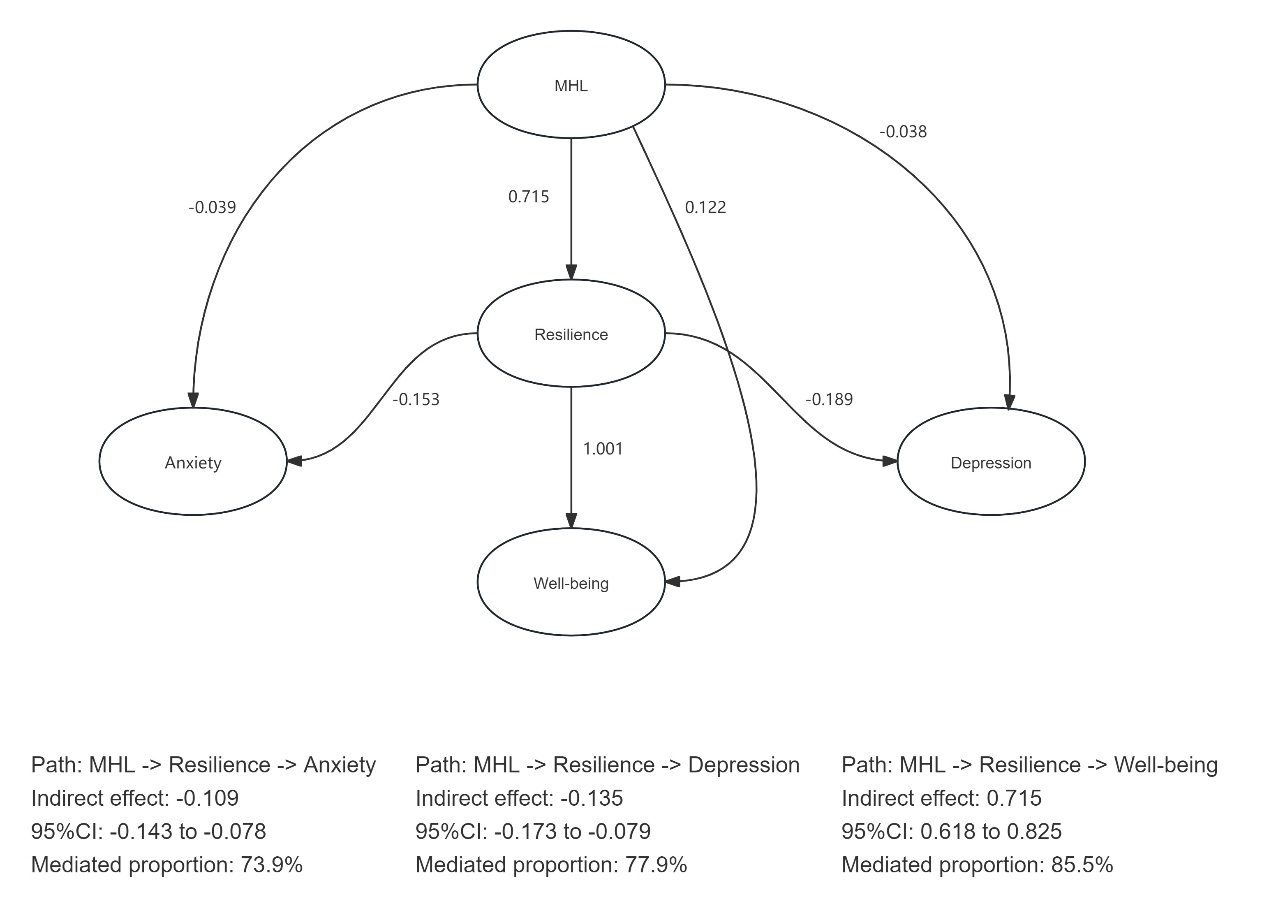


Supplementary Figure 1 Path diagram of SEM analysis. MHL, mental health literacy.
